# The effect of progressive and individualised sport-specific training on the prevalence of injury in football and handball student athletes: a randomised controlled trial

**DOI:** 10.3389/fspor.2023.1106404

**Published:** 2023-06-06

**Authors:** Cathrine Nyhus Hagum, Espen Tønnessen, Jonny Hisdal, Shaher A. I. Shalfawi

**Affiliations:** ^1^ Department of Education and Sports Science, University of Stavanger, Stavanger, Norway; ^2^ Faculty of Health Sciences, Kristiania University College, Oslo, Norway; ^3^ Department of Vascular Surgery, Oslo University Hospital, Oslo, Norway

**Keywords:** student athletes, injury prevalence, load management, communication and coordination, progressive overload

## Abstract

**Objective:**

To evaluate the effectiveness of communication and coordination combined with designing a progressive and individualised sport-specific training program for reducing injury prevalence in youth female and male football and handball players transitioning to a sports academy high school. An additional aim was to investigate the characteristics of the reported injuries.

**Methods:**

Forty-two Norwegian athletes were randomised into an intervention or control group. Mean age, height, weight and BMI was 15.5 ± 0.5 years, 178.6 cm ± 6.3 cm, 71.3 ± 9.8 kg, 22.3 ± 2.7 BMI for the intervention group (IG) (*n* = 23), and 15.4 ± 0.5 years, 175.6 cm ± 6.6 cm, 67.1 ± 9.8 kg, 21.7 ± 2.4 BMI for the control group (CG) (*n* = 19). During the summer holiday, the intervention group received weekly progressive, individualised sport-specific training programs and weekly follow-up telephone calls from the researchers. All athletes completed a baseline questionnaire and a physical test battery. Training data and injuries were recorded prospectively for 22 weeks using the Oslo Sports Trauma Research Center Questionnaire on Health Problems (OSTRC-H2). A two-way chi-square (*χ*^2^) test of independence was conducted to examine the relationship between groups and injury.

**Results:**

Average weekly prevalence of all injuries was 11% (95% CI: 8%–14%) in IG and 19% (95% CI: 13%–26%) in CG. Average weekly prevalence of substantial injuries was 7% (95% CI: 3%–10%) in IG and 10% (95% CI: 6%–13%) in CG. The between-group difference in injuries was significant: *χ*^2^ (1, *N* = 375) = 4.865, *p* = .031, *φ* = .114, with 1.8 times higher injury risk in CG vs. IG during the first 12 weeks after enrolment.

**Conclusions:**

For student athletes transitioning to a sports academy high school, progressive individualised, sport-specific training programs reduced the prevalence of all-complaint injuries following enrolment. Clubs and schools should prioritise time and resources to implement similar interventions in periods where student athletes have less supervision, such as the summer holidays, to facilitate an optimal transition to a sports academy high school.

## 1. Introduction

Several injury prevention programs are used in teams sports, such as the FIFA 11+ warm-up programme (1, 2), and Sportsmetrics (3), while other programs target specific injury locations, such as the shoulder (4) and hamstring (5). Injury prevention is complex, and requires consideration of the multiple factors contributing to injury (6). Therefore, practitioners should collaborate in a multimodal injury prevention process (6), and load management through individualised training programs has been suggested as a preventive measure (7).

Following sports academy high school enrolment, elite youth athletes are at high risk of injury (8–10). Rapid increases in training load can increase the risk of injury (11), with almost 60% of non-contact injuries occurring during the transition back into training following a period of inactivity (10). If the applied physical load is substantially higher than the athlete's physical capacity, tissue tolerance will be exceeded and injury can occur (12). Previous research has reported high injury prevalence in youth elite handball and football players (7, 9, 13, 14). Injuries and absence from training and matches can impede individual development (14, 15), and potentially have negative psychological effects (15–17). Furthermore, injuries negatively impact the team and individual athletic success (18). This study therefore aimed to evaluate the effect of a progressive, individualised sport-specific training program with weekly follow-up on injury prevalence in football and handball players transitioning to a sports academy high school. An additional aim was to investigate the characteristics of the reported injuries.

## 2. Materials and methods

### 2.1. Study design and recruitment

The study was conducted as a 22-week randomised controlled trial from June to November 2021. Student athletes were recruited from three sports academy high schools in Norway. Student athletes who applied and were accepted to the selected schools in 2021 were eligible for inclusion. Other inclusion criteria were that they played football or handball, were born in 2005, and could perform a physical test battery without pain (i.e., injury free). Eligible participants were randomly allocated to an intervention group (IG) or control group (CG) using a computer-generated, random allocation sequence generated by two of the researchers in this study. Randomisation was stratified by sex, sport, and performance level (i.e., physical fitness, motor performance, sport-specific and skills). The athletes’ coaches (school, club and regional) took part in assessing and ranking each participant based on their level of performance prior to randomisation.

The participants and their guardians were informed of the experimental risks and signed an informed consent document prior to the investigation. This study was registered at Norwegian Social Science Data Services (NSD) (Project number: 836079) and approved by the West Norwegian Regional Committees for Medical and Health Research Ethics (REK) (project number: 54584).

### 2.2. Participants

**Figure 1:**
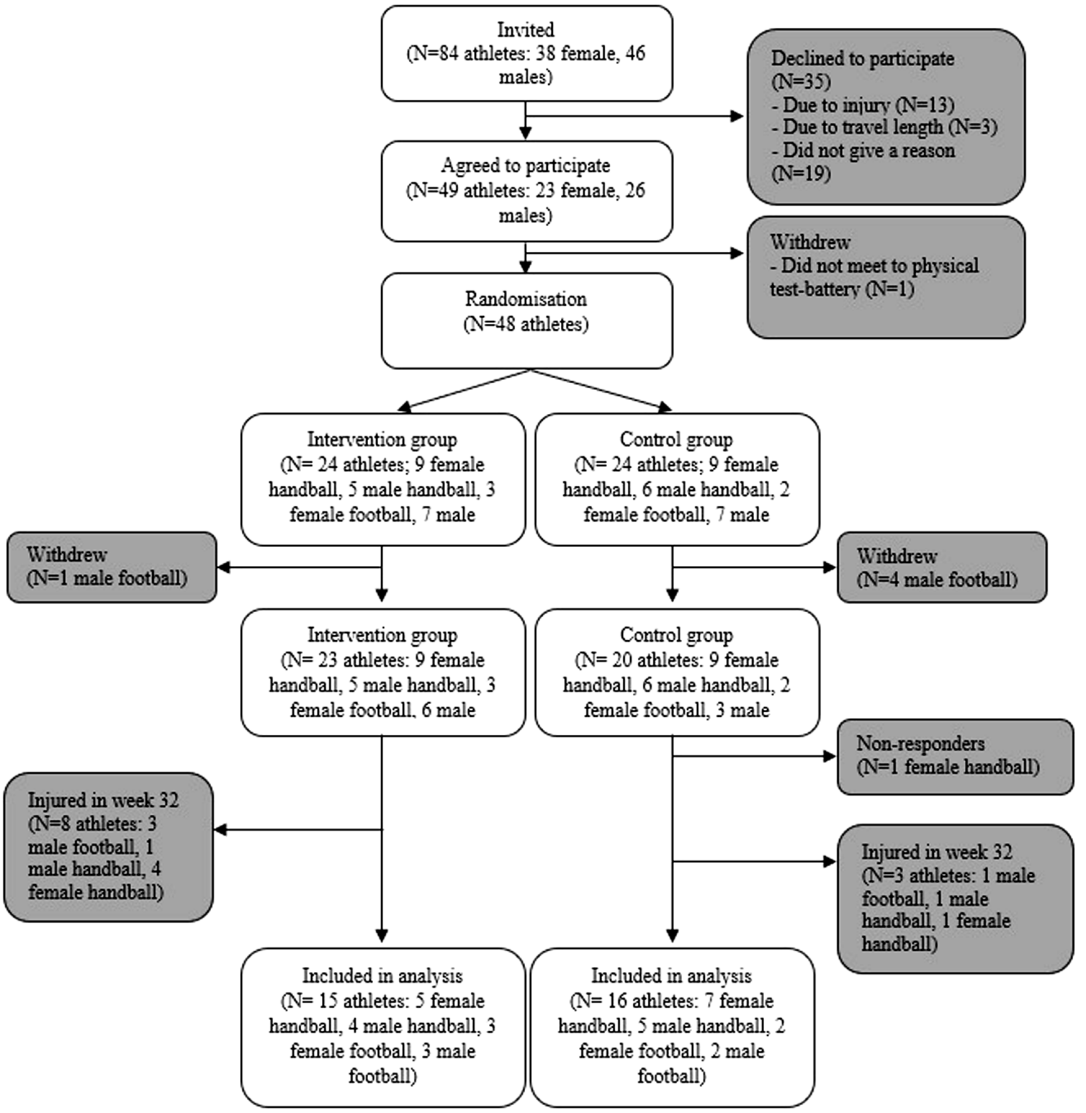
Participant flow throughout the study. The final analyses did not include athletes reporting an injury the week prior to enrolment (week 32).

Out of 84 eligible athletes who applied to the selected schools, 49 agreed to participate. Six participants withdrew, and one participant stopped responding, leaving a total sample of 42 participants (22 females, 20 males). Of these, 64% were on regional and/or national teams, and all competed for sports clubs not affiliated with their sport's high schools. The football players were distributed among five sports clubs, while handball players were distributed among 11 sports clubs. Baseline characteristics were collected in May 2021 using an electronic questionnaire (Survey Xact) (19), including information about the participant's school, type of sport, and training history for the past two weeks. Figure 1 illustrates the participant flow.

### 2.3. Procedure and intervention

**Figure 2:**
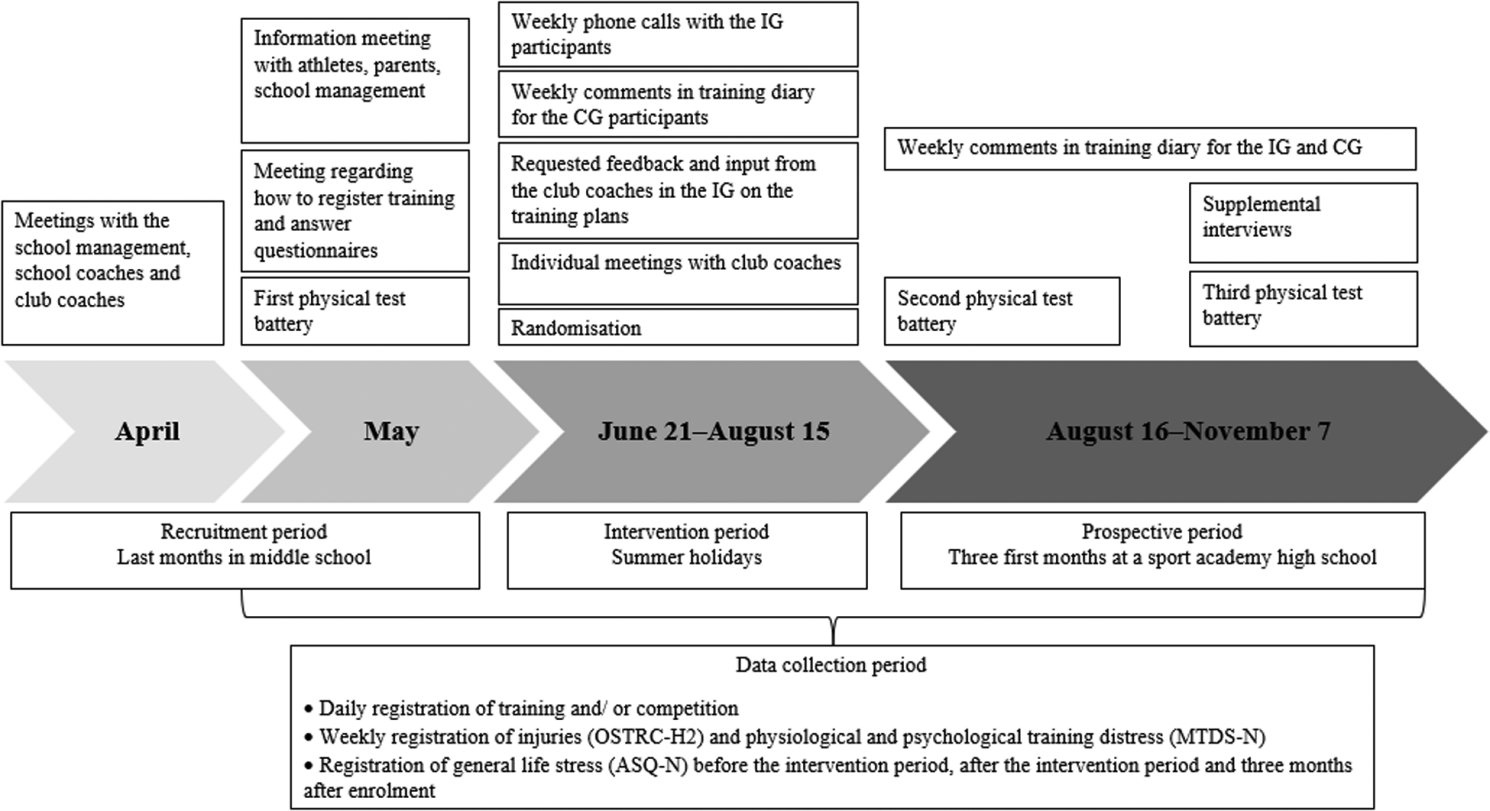
Timeline of the study.

To improve compliance, all participants, guardians, and coaches were invited to a meeting where information about the study purpose, procedures, and timeline was provided. Figure 2 illustrates when the meetings, data collection and intervention took place. All participants received information about the physical test battery one week before completion. On the day of testing, the research team demonstrated the different tests and participants got to try the different exercises before registration. During the 8-week transition period (i.e., the summer holiday from mid-june to mid-August), participants in IG and CG received an injury prevention program and were instructed to perform the program three times a week. In addition to the injury prevention program, IG received weekly progressive individualised sport-specific training programs during the 8-week transition period. The CG did not receive a progressive individual sport-specific training and were asked to do their normal training. After the 8-week transition period, all participants did their normal training (i.e., IG did not receive progressive individualised sport-specific training programs and none of the groups were required to complete the injury prevention program).

### 2.4. The progressive individual sport-specific training programs

Prior to the intervention period (8-week transition), communication in the form of individual meetings were conducted with the athletes' coaches to collect information about individual players' current training load, injury history, club training during the summer and expected training load when starting at the sports academy high school. This information was used to prepare the first weekly training program. Each subsequent weekend, two of the researchers is this study completed phone calls with each of the players in the IG, where information about their week was collected (i.e., how they felt, if they had done all the prescribed training, which changes had been made to the program, how did they tolerate the training program, available training facilities, and their vacation plans). Based on the communication with the student athlete, a new training program with a progressive stimulus for the next week was created and emailed to the athlete, guardians and coaches. Halfway through the intervention (week 4), all the coaches were sent an email requesting feedback and input on the training plans. If coaches, athletes, or guardians had any questions, they could contact two of the researchers via SMS or telephone anytime during the study.

The training plans were developed by an expert in sports science with experience working with Olympic and World champions from various sports (e.g., swimming, handball, track and field, cross-country skiing). The principle of progressive overload was used by increasing the training load gradually when the athlete had adapted to a specific training load or stimuli (20, 230). A form of fluctuating overload was applied (20, 228–229). Using evidence-based practice, the training plans were developed focusing on tissue-specific strength and tissue-specific stress and strain to improve the participant's tolerance for sport-specific training (6, 21). Furthermore, participants had access to a digital platform where the researchers published videos and other resources on how to perform the different exercises in their weekly training plan. An example of a training plan for a handball and football player can be found in the supplementary material (Supplementary Figure S1).

The expert developing the training plans adopted a holistic view (e.g., took into account social factors, family obligations, and a need for mental regeneration) when defining individual training variables (e.g., frequency, volume, intensity) and modalities of the exercise intervention (22). Other factors carefully considered during the eight weeks of training prescription were player training background, accumulated training, match exposure, injury history, player's personality and preferences, and off-season length (22). The program was not always done exactly as prescribed. However, with weekly follow-ups by the researchers, it was possible to make adjustments to ensure progressive overload and appropriate distribution of physical or sport-specific training. We believe that weekly follow-ups ensured high compliance.

### 2.5. Training diary and injury reporting

All participants recorded their training using an electronic training diary (www.bestr.no, Lørenskog, Norway). They reported training duration for handball or football activities, strength training, endurance, sprint and jump training, stretching, and injury prevention. Rating of perceived exertion (RPE) was also reported in the electronic training diary and was collected using the modified Borg category ratio RPE scale (23). Session RPE (sRPE) was derived by multiplying RPE by session duration (minutes). In addition, the participants reported weekly physiological and psychological training distress in the electronic training diary by using the Norwegian version of the Multicomponent Training Distress Scale (MTDS-N) (24). Three times during the data collection (i.e., before the intervention period, after the intervention period and after three months after enrolment), the participants reported general life stress in the electronic training diary by using the Norwegian version of The Adolescent Stress Questionnaire (ASQ-N) (25). In week 20, one of the researchers conducted individual meetings with participants to review the registered training and ensure that data were being reported correctly. Due to its scope, the data collected from the physical test batteries, MTDS-N and ASQ-N are not included in the results.

The Oslo Sports Trauma Research Centre Questionnaire on Health Problems (OSTRC-H2) was used to record injuries (26). Players received the questionnaire electronically every Friday and were instructed to report health problems for the previous seven days. Participants were instructed to report all complaints, irrespective of their consequences for sports participation. If a participant answered “full participations without any health problems” (first answer option), all further questions were redundant, and a total severity score of 0 was assigned. If a participant answered “could not participate due to a health problem,” questions 2–4 were redundant, and a total severity score of 100 was assigned. If a health problem was reported, the athlete was asked to report additional information, such as the type of the problem and its location or main symptoms (27). The location was categorised according to the OSTRC Questionnaire on Health Problems (27). The mode of onset was collected according to the most recent IOC consensus (28). If a player registered alternative two or higher (i.e., moderate to severe reduction or inability to participate) in question 2 (training volume) or 3 (performance), the health problem was registered as substantial. Non-responders received a personal SMS reminder every Monday. At the end of the study, in-person interviews were conducted with each participant to supplement missing data and verify the collected data's accuracy.

### 2.6. Outcome measures

The primary outcome measure was weekly prevalence of injuries registered after enrolment. An injury was defined as a response above the minimum value on at least one of the four key questions in the OSTRC-H2 (i.e., all complaint definition) (27). Only injuries resulting directly from participation in a competition or from training of fundamental sporting skills were included (28). The secondary outcomes included injury location and mode of onset. Substantial injuries were defined as injuries leading to a moderate or severe reduction in training volume or performance or inability to participate (27).

### 2.7. Statistical methods

All statistical procedures were performed using IBM SPSS statistics V.27.0. Continuous variables are presented as mean (M) and standard deviation (SD). Ordinal or categorical variables are presented as percentages. Independent sample *t*-tests were performed to investigate differences in baseline characteristics, sRPE and training volume (hours). Injury prevalence was calculated by dividing the number of athletes reporting an injury or a substantial injury by the total number of respondents in each group (29). For all injury prevalence variables, 95% confidence intervals (CI) were calculated. A two-way chi-square (*χ*^2^) test of independence was conducted to examine the relationship between groups and injury. Period (week 11–14; 15–18; 19–22) was used as a stratifying variable. Fisher's exact test was used to reduce the chance of making a Type I error (30, 290), and the statistical significance level was set at *p* < 0.05 for all analyses. The effect size was evaluated using the phi coefficient (*φ*). A value of 0.1, 0.3, and 0.5 indicated small, medium, and large associations between groups, respectively (31). Relative risk (RR) and corresponding 95% CI was also calculated. No data imputations were made for missing data. All analyses were performed according to the intention-to-treat principle. One participant stopped responding during the project and could, for this reason, not be included in the final analysis. In addition, the final analyses did not include athletes reporting an injury the week prior to enrolment.

### 2.8. Sample size

The sample size was based on the number of observations per group using the sequential Bayes Factor Design Analysis (BFDA) (32–34), calculating the number of observations required to estimate a difference that is 80% true and a nondifference that is 80% true. To avoid underestimating the sample number of observations, we used an effect size of *d* = 0.2 with a small symmetric decision boundary of 6 (i.e., moderate evidence) (35). All calculations were conducted using the BFDA app (33) at http://shinyapps.org/apps/BFDA/. The results of the sequential BFDA indicated that for the difference to be 80% correct using the default Prior on Effect Size, this required ≥235 observations, and ≥120 observations for the none difference to be 80% correct. In this study, the OSTRC-H2 observations were 727 (376 from IG and 351 from CG). A total of 6,864 training session observations were registered (3,981 from IG and 2,883 from CG), and sRPE was registered for 6,565 training sessions (3,836 from IG and 2,729 from CG). Finally, 4,095 exposure hours were recorded (2,406 for IG and 1,689 for CG).

## 3. Results

Mean age, height, and weight was 15.5 ± 0.5 years, 178.6 cm ± 6.3 cm, 71.3 ± 9.8 kg for IG (*n* = 23), and 15.4 ± 0.5 years, 175.6 cm ± 6.6 cm, 67.1 ± 9.8 kg for CG (*n* = 19) (Table 1). A total of 924 OSTRC-H2 questionnaires were sent to the participants for 22 weeks, and 727 were completed, resulting in a response rate of 79%. The response rate in the IG was 74%, while the response rate in the CG was 84%. After completing the supplemental interviews, 100% of the questionnaires were answered. Table 2 provides a summary of the training conducted during the intervention period.

**Table 1:** Baseline characteristics of the intervention and control group (*n* = 42)^1^.

	Intervention (*n* = 23)		Control (*n* = 19)	
Age (years)	15.52 ± 0.51		15.37 ± 0.50	
Sex^2^ (*n*)	F (12)	M (11)	F (10)	M (9)
Type of sport^3^ (*n*)	HB (9)	HB (5)	HB (8)	HB (6)
	FB (3)	FB (6)	FB (2)	FB (3)
	F	M	F	M
Height (cm)	174.17 ± 4.04	183.45 ± 4.37	172.40 ± 5.13	179.22 ± 6.46
Weight (kg)	67.30 ± 4.05	75.59 ± 12.34	64.32 ± 8.10	70.21 ± 10.95
CMJ (cm)	29.33 ± 3.19	36.03 ± 5.68	29.80 ± 3.62	39.54 ± 5.86
Sit-ups (reps)	15.08 ± 6.64	20.36 ± 6.67	15.50 ± 5.04	20.89 ± 6.94
30 meter (sec)	4.88 ± .18	4.44 ± .23	4.86 ± .20	4.42 ± .19
Throwing/shooting velocity (km/t)	84.25 ± 8.66	104.09 ± 9.79	79.00 ± 6.04	99.78 ± 8.77
Bleep test (m)	1,495.00 ± 254.29	2,100.00 ± 337.52	1,492.00 ± 204.44	2,142.22 ± 216.44

^1^ Data are presented as M ± SD unless otherwise specified.

^2^ F, female; M, male.

^3^ HB, handball; FB, football.

**Table 2:** Mean training volume (hours) during the intervention period (week 2–9).

	Control group			Intervention group		
Type of training	All (*n* = 19)	Handball (*n* = 14)	Football (*n* = 5)	All (*n* = 23)	Handball (*n* = 14)	Football (*n* = 9)
Total	7.8 ± 2.4	7.9 ± 2.4	7.7 ± 2.9	10.7 ± 1.9*	10.5 ± 2.0*	11.1 ± 1.8*
Specific^1^	2.4 ± 1.3	1.9 ± 0.6	4.1 ± 1.6	3.7 ± 1.7*	2.7 ± 1.1*	5.1 ± 1.4
Physical	3.2 ± 2.2	3.8 ± 2.1	1.2 ± 1.3	4.4 ± 1.0*	4.8 ± 0.7	3.7 ± 0.9*
Injury prevention	0.8 ± 0.4	0.7 ± 0.3	1.2 ± 0.5	1.1 ± 0.3*	1.2 ± 0.3*	1.0 ± 0.3
sRPE^2^	40.7 ± 12.8	42.8 ± 10.5	33.1 ± 18.9	50.6 ± 10.5*	53.6 ± 10.1*	46.0 ± 10.0

^1^ Sport-specific training performed individually or with the team. Physical training includes endurance, strength, speed/velocity, and jump training. Total training is the sum of specific, physical, injury prevention, warm-up and other training.

^2^ Weekly total session rating of perceived exertion during the intervention period (mean ± SD).

* Statistically significant difference from CG (*p* < 0.05).

The athletes' mean training volume and weekly sRPE after enrolment are presented in Table 3. There were no significant differences in training volume between IG and CG after enrolment, other than for injury prevention, where IG (all) and IG (football) performed less injury prevention compared to CG (all) and CG (football). Further, weekly sRPE in weeks 14–18 was notably higher in IG (all) and IG (football) compared to CG (all) and CG (football).

**Table 3:** Mean training volume (hours) during the 12 first weeks at sports academy high school.

		Control group			Intervention group		
Period (week)	Type of training	All (*n* = 16)	Handball (*n* = 12)	Football (*n* = 4)	All (*n* = 15)	Handball (*n* = 9)	Football (*n* = 6)
11–14	Total	12.3 ± 3.3	12.3 ± 3.6	12.1 ± 2.3	11.7 ± 1.8	10.7 ± 1.4	13.1 ± 1.1
	Specific^1^	6.1 ± 1.5	6.3 ± 1.5	5.4 ± 1.3	6.4 ± 2.7	5.4 ± 1.7	7.9 ± 3.4
	Physical	2.4 ± 1.0	2.5 ± 1.2	2.2 ± 0.4	3.2 ± 2.7	3.8 ± 3.3	2.2 ± 0.8
	Injury prevention	0.4 ± 0.4	0.4 ± 0.4	0.1 ± 0.1	0.1 ± 0.1*	0.1 ± 0.1*	0.1 ± 0.1
	sRPE^2^	50.3 ± 16.1	52.4 ± 17.1	41.5 ± 7.2	52.5 ± 9.4	52.9 ± 11.7	51.9 ± 6.1
15–18	Total	11.1 ± 2.5	11.2 ± 2.4	10.9 ± 3.3	12.2 ± 2.5	11.4 ± 1.7	13.3 ± 3.3
	Specific	5.7 ± 1.3	5.6 ± 1.4	6.2 ± 1.1	6.6 ± 2.8	5.3 ± 1.0	8.5 ± 3.7
	Physical	2.5 ± 1.1	2.6 ± 1.2	2.5 ± 1.1	2.9 ± 2.0	3.1 ± 2.4	2.8 ± 1.2
	Injury prevention	0.2 ± 0.2	0.3 ± 0.2	0.0 ± 0.0	0.1 ± 0.1	0.1 ± 0.1*	0.1 ± 0.2
	sRPE	44.8 ± 10.7	47.6 ± 9.9	33.3 ± 3.8	55.1 ± 10.0*	55.3 ± 7.5	54.8 ± 13.7*
19–22	Total	10.5 ± 3.1	10.5 ± 3.1	10.4 ± 3.6	10.9 ± 2.3	10.4 ± 2.6	11.7 ± 1.7
	Specific	4.7 ± 2.1	4.7 ± 2.4	4.6 ± 1.2	5.6 ± 2.5	5.1 ± 1.6	6.4 ± 3.4
	Physical	3.1 ± 1.7	3.2 ± 1.9	2.0 ± 0.6	2.8 ± 1.8	3.3 ± 2.2	2.1 ± 0.8
	Injury prevention	0.3 ± 0.5	0.4 ± 0.6	0.0 ± 0.0	0.1 ± 0.3	0.2 ± 0.4	0.1 ± 0.1
	sRPE	42.8 ± 13.3	44.0 ± 14.5	38.0 ± 6.8	48.8 ± 7.4	48.6 ± 7.9	49.1 ± 7.4

^1^ Specific training consists of sport-specific training performed individually or with the team. Physical training includes endurance, strength, speed/velocity, and jump. Total training consists of specific, physical, injury prevention, warm-up and other training (e.g., volleyball at school, tennis during vacation etc).

^2^ Weekly total sRPE during the intervention period (mean ± SD).

* Statistically significant difference from CG (*p* < 0.05).

### 3.1. Intervention effect on injury prevalence in groups

**Figure 3:**
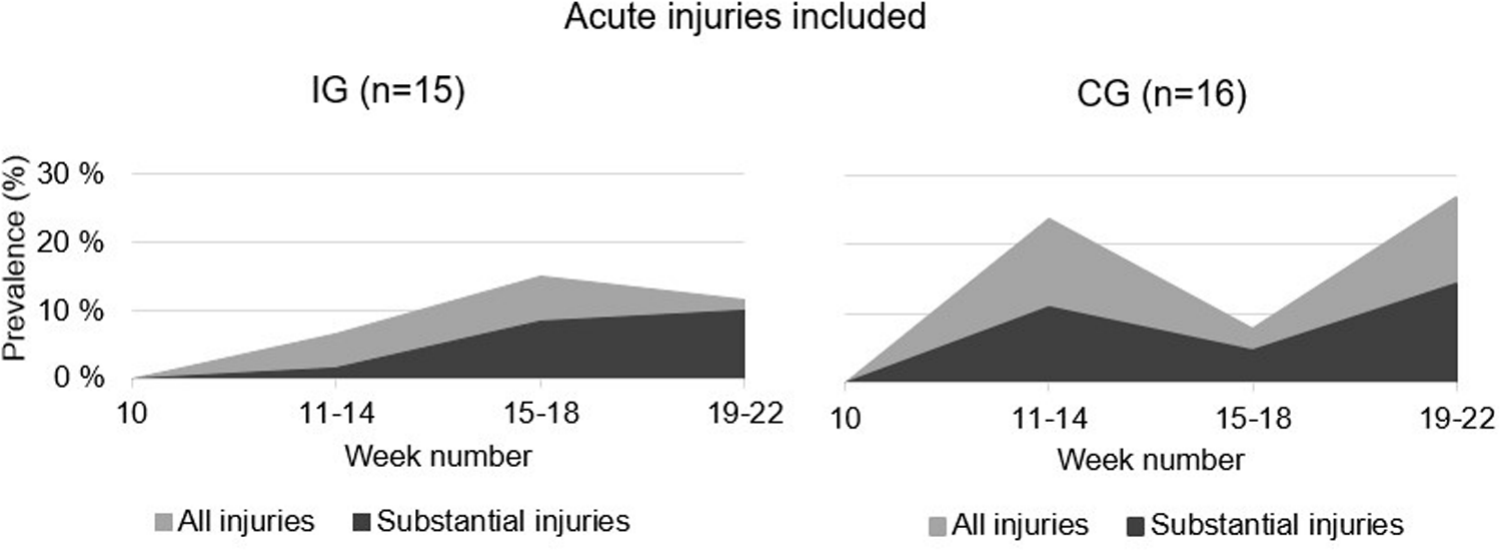
Point prevalence proportion of all injuries (light area) and substantial injuries (dark area) in IG and CG 12 weeks after enrolment into a sports academy high school.

The average weekly prevalence of all injuries was 11% (95% CI: 8%–14%) in IG and 19% (95% CI: 13%–26%) in CG. The average weekly prevalence of substantial injuries was 7% (95% CI: 3%–10%) in IG and 10% (95% CI: 6%–13%) in CG. The prevalence measures are illustrated in Figure 3. The proportion of athletes reporting an injury after enrolment differed between groups: *χ*^2^ (1, *N* = 375) = 4.865, *p* = .031, *φ* = .114, indicating a small effect size. The RR was 1.75 (95% CI: 1.05–2.89). When dividing the 12 weeks into three periods, the proportion of athletes who reported an injury differed by group in weeks 11–14: *χ*^2^ (1, *N* = 125) = 6.904, *p* = .012, *φ* = .235 and in weeks 19–22: *χ*^2^ (1, *N* = 124) = 4.402, *p* = .042, *φ* = .188. The RR was 3.57 (95% CI: 1.26–10.17) and 2.28 (95% CI: 1.02–5.10), respectively. There were no significant group differences in weeks 15–18. The injury prevalence in groups by sport can be found in the supplementary material (Supplementary Figure S2).

### 3.2. Characteristics of the reported injuries

**Figure 4:**
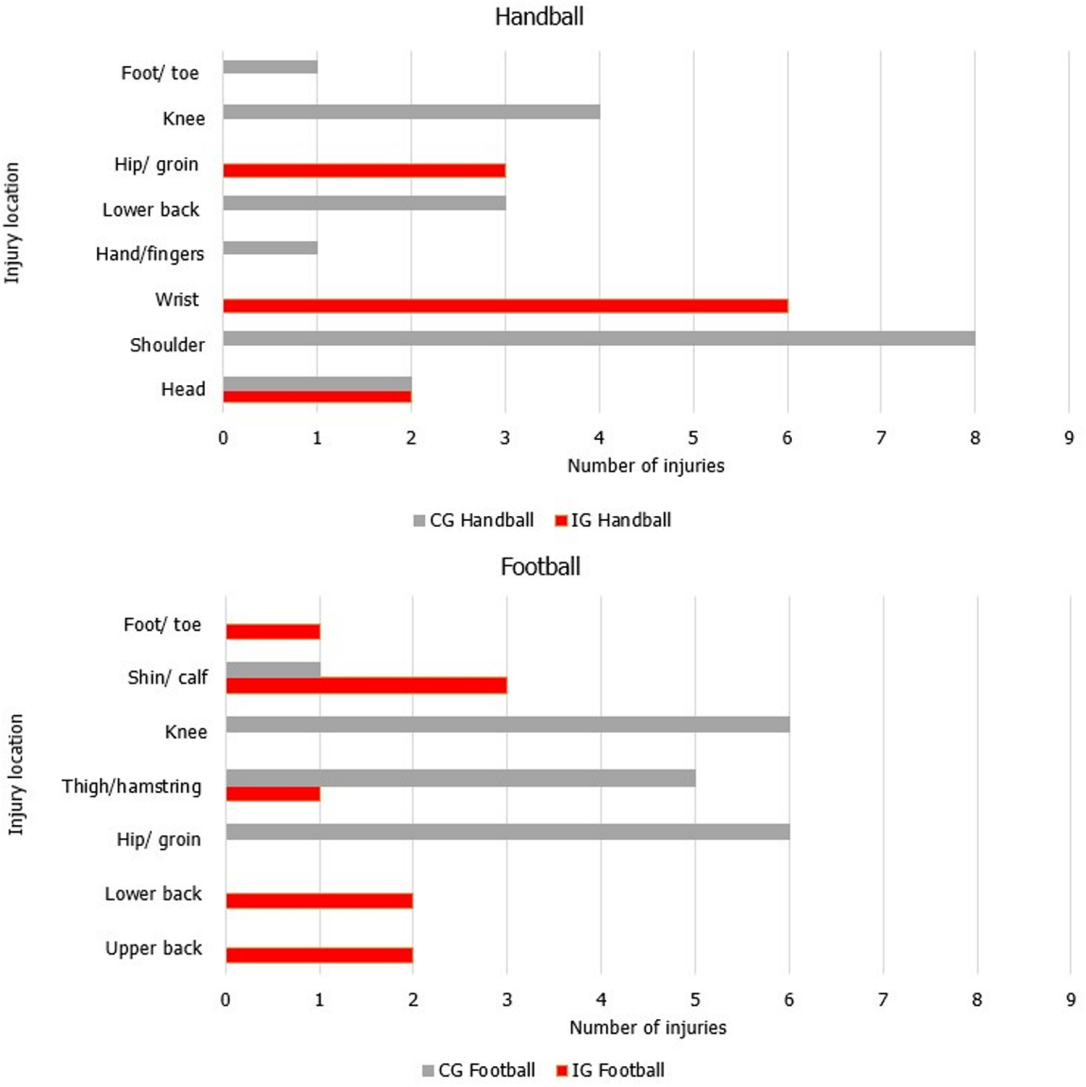
Injury location for the reported injuries after enrolment in the intervention group and the control group in handball and football players of both sexes. The same injury could be reported in subsequent weeks by an athlete.

**Figure 5:**
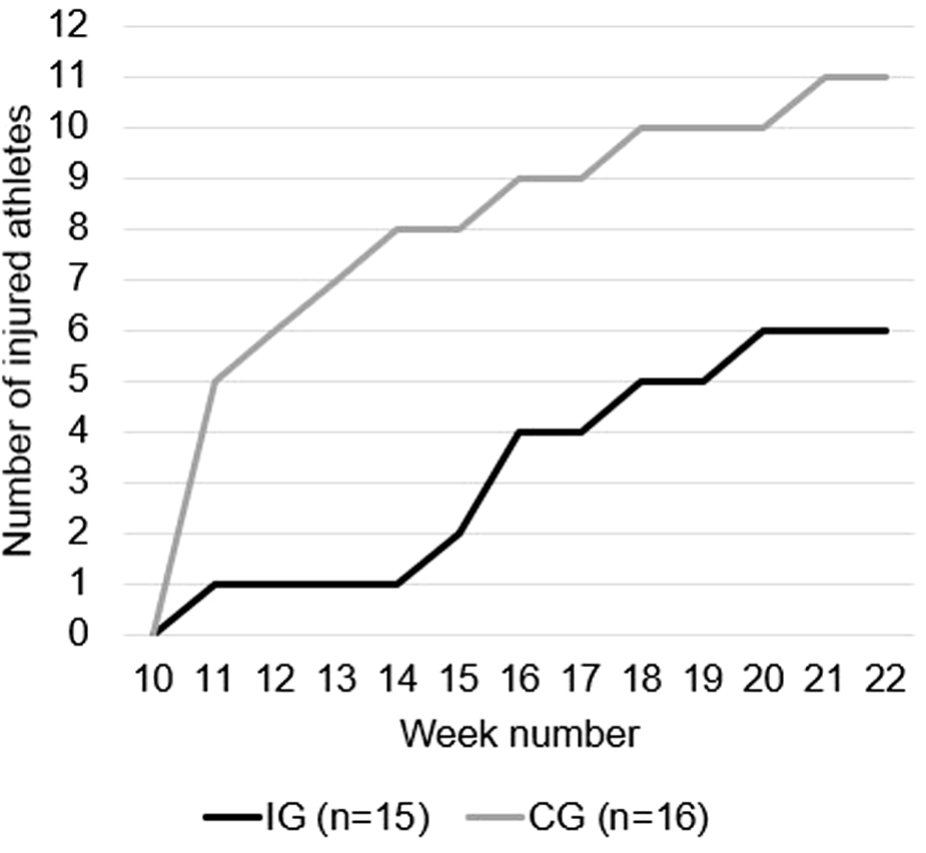
Cumulative incidence cases show the number of athletes sustaining an injury weekly in IG and CG after enrolment into sports academy high school.

After enrolment, 20 injuries were reported by the 15 athletes in the IG (50% were acute, 15% were repetitive with a sudden onset, and 35% were repetitive with a gradual onset). By the 16 athletes in CG, 37 injuries were reported (24% were acute, 43% were repetitive with a sudden onset, and 33% were repetitive with a gradual onset). The location of the injuries is shown in Figure 4. Figure 5 shows the cumulative number of injury incidents each week after enrolment, illustrating the number of athletes with at least one injury.

## 4. Discussion

The main findings of the present study are that average weekly prevalence of all injuries was 11% (95% CI: 8%–14%) in IG and 19% (95% CI: 13%–26%) in CG. Average weekly prevalence of substantial injuries was 7% (95% CI: 3%–10%) in IG and 10% (95% CI: 6%–13%) in CG. The athletes in CG had a 1.8 times higher risk of injury after enrolment compared to IG.

### 4.1. Intervention effect on injury prevalence in groups

Injury prevalence was lower in our study compared to previous studies in a comparable sample (7, 9). This could be due to the fact that the present study included only injuries resulting directly from participation in a competition or training in the sport's fundamental skills over a short period (12 weeks) compared to Bjørndal, et al. (9) and Moseid, et al. (7) who included all injuries over a more extended period (∼33 and ∼22 weeks, respectively). In addition, both IG and CG in our study completed an injury prevention program three times a week during the summer.

In the current study, IG experienced more acute injuries than CG. A higher proportion of acute injuries correspond with previous findings in youth team athletes (7, 9, 36–38). However, athletes in CG were more prone to repetitive injuries. This is an important finding since acute injuries occur relatively frequently due to the nature of the activities (13). In football and handball, players perform multiple intense movements in different directions (accelerations, decelerations, side-cutting, jumping, and landing) and are involved in tackling situations (39–41), increasing the risk of injury (42, 43). Hence, acute injuries are difficult to prevent with the progressive individualised sport-specific training programs that IG received. We believe that such training programs are primarily preventative against injuries occurring from a gradual accumulation of low-energy transfer over time (e.g., bone stress injury) or from a combination of acute and gradual onset (e.g., repetitive training resulting in tendon weakness, presenting acutely as a tear from acceleration forces applied during a sprint) (28).

After enrolment, CG had 1.8 times higher injury risk compared to IG. When dividing the first 12 weeks into three periods, CG had a 3.5 and 2.3 higher risk of becoming injured in the first and last four weeks after enrolment, respectively. As shown in Figure 5, 40% of athletes in IG became injured, whereas ∼69% became injured in CG. Injuries were distributed between several different athletes in the groups, particularly in CG. Since alterations resulting from previous injuries may overload other structures not involved in the initial injury (6), sustaining an injury increases the risk of a recurrence of both the original injury as well as subsequent injury of any type (44, 45). However, a gradual, and systematic increase in training load during the summer (Table 2) appears to contribute to a safe progression in training load, improving players' tolerance to training towards the end of the summer. This in turn can reduce injury risk and enhance performance (46, 47).

### 4.2. Characteristics of the reported injuries

In handball athletes, wrist and shoulder/collarbone injuries were the most frequently reported in IG and CG, respectively, with the second most frequently reported injury being the knee for CG (Figure 4). The wrist and shoulder/collarbone injuries could be gradual onset injuries caused by the repetitive throwing motion in handball (37). However, 100% of the wrist injuries were categorised as acute. For the shoulder/collarbone injuries, 75% of the injuries were categorised as repetitive with a sudden onset, while 15% were categorised as repetitive with a gradual onset. The OSTRC shoulder injury prevention programme has been shown to reduce the prevalence of shoulder injuries when used during warm-up in elite handball players (4). No shoulder or knee injuries were observed in IG, indicating that the individualised training program involving strength training, throwing with medicine and tennis balls, handball drills, sprints, agility and jump exercises during the summer holiday might be effective in preventing injuries in these locations. Table 2 indicates that CG lacked sport-specific training during the summer, resulting in greater injury risk when performing technically demanding skills after enrolment.

In football players, the most common injury location was the shin/calf for IG, followed by the lower back and ribs/upper back. In CG, injuries to the hip/groin and knee were the most frequent, followed by the thigh. No knee injuries occurred in IG. The injury locations in CG are comparable with previous research reporting that the thigh, knee, ankle, and hip/groin are the most frequently injured locations in youth elite football players (38, 48–51). After enrolment, no knee injuries occurred in IG. The injury pattern in IG differs from other studies in these age groups (14). A possible explanation is the low number of athletes and injuries in the current study. Previous research has shown that including the Nordic Hamstrings exercise in injury prevention programmes reduces the risk of hamstring injuries (5). In addition, the Copenhagen Adductor exercise might function to prevent groin injuries (52, 53). A combination of these exercises does also seem to be beneficial (54, 55). However, disregarding the effectiveness of separate exercises or combinations of exercises, we believe a comprehensive and holistic training program including specific football exercises, strength training, sprints, agility, and jump exercises might prevent common injuries in football, suggesting that specificity is a vital training principle to prevent injuries. Still, we acknowledge that training load is only one of many contextual factors that must be considered when managing athlete injury and readiness to perform (47, 56).

### 4.3. Methodological considerations and limitations

To our knowledge, this is the first study investigating this population in this particular transition period in a Scandinavian context. A strength of this study is the high compliance with the training programs and the high response rates for training data and the OSTRC-H2. To minimise the survey burden, we followed the 2020 update of the OSTRC-H2 (28), where the survey ends if the player reports “full participation without health problems” for the first question. However, the OSTRC-H2 is not a validated approach for adolescent population (26), and must be considered as a limitation in the current study. The age group and study context should be considered when adapting and applying the OSTRC-H2 to adolescents (57). In addition, athlete-self reported data may have resulted in inaccurate reporting.

Another limitation of the study is the low participation rate. Out of 84 eligible athletes, only 49 agreed to participate (58% of eligible players) and only 42 completed the study (50% of 84 eligible players), which reduced effective sample size, statistical power and increased the risk for selection bias (58). Due to the small sample size, we used the sequential BFDA (32–34). The sample was also obtained using a convenience sampling method, limiting generalisability. The intention-to-treat principle could introduce selection bias due to the participants not being included in the final analysis. Lastly, we did not account for previous injuries in the randomisation. The objective of randomisation is to have balanced groups (59), but with the small sample size in the current study, it might be a chance that the proportion of athletes with previous injuries could differ due to random bias, which could have significant effects on the results.

### 4.4. Practical implications

As a coach, it can be challenging to individualise training for a team athlete, particularly during longer breaks from organised club training. Close supervision and individualised training programs during the summer holidays should not be an additional task left solely to the coach, but should be prioritised by the club and school, and given extra resources. Implementing this type of intervention also requires close communication, not only with the athletes themselves but also with other key persons such as guardians, coaches, the school, and potentially a medical support system. An effective injury prevention strategy can increase sports participation and performance development and should therefore be prioritised.

## 5. Conclusion

The results indicated a reduction in the prevalence of injuries in IG compared to CG. Managing training load with a holistic perspective and ensuring a progressive overload in athletes during the summer holidays appears to be an effective intervention to prevent injuries after enrolment in football and handball athletes of both sexes. The results of this study can increase awareness of the importance of implementing measures in periods where the club and school have reduced organised activities for the athletes. Someone must take responsibility for making plans and following up on the athlete when they are not part of organised training activity, such as during the summer holidays. Future studies should include larger sample size and possible confounders like sleep, nutrition and hydration.

## Data Availability

The raw data supporting the conclusions of this article will be made available by the authors, without undue reservation.
