# Supplementary figures and images for: The effect of progressive and individualised sport-specific training on the prevalence of injury in football and handball student athletes: a randomised controlled trial
Source: Front Sports Act Living. 2023 Jun 6;5:1106404. doi: 10.3389/fspor.2023.1106404 (PMC10279870; doi:10.3389/fspor.2023.1106404)

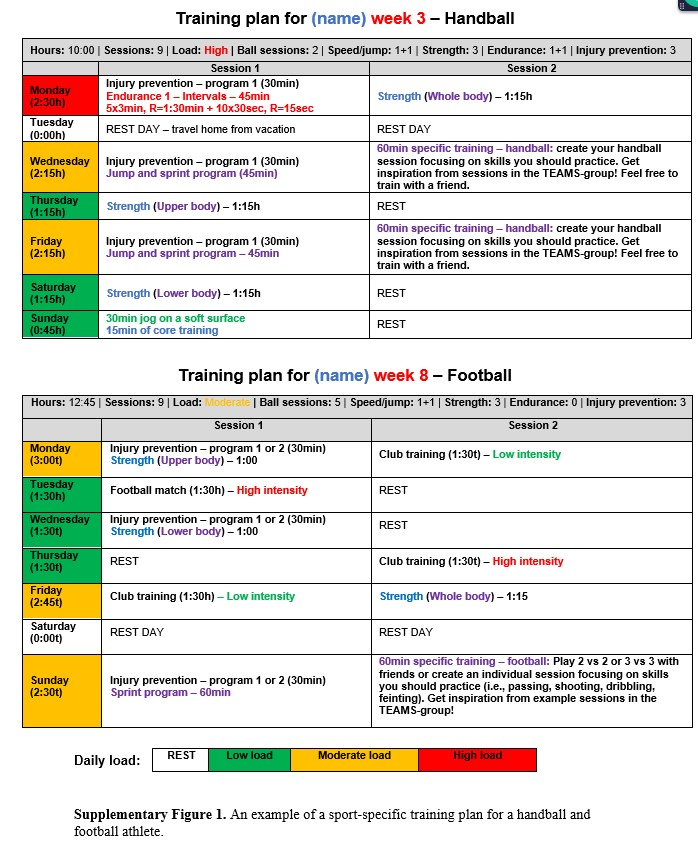

Supplement: Supplementary file 1 [file Image1.tiff]

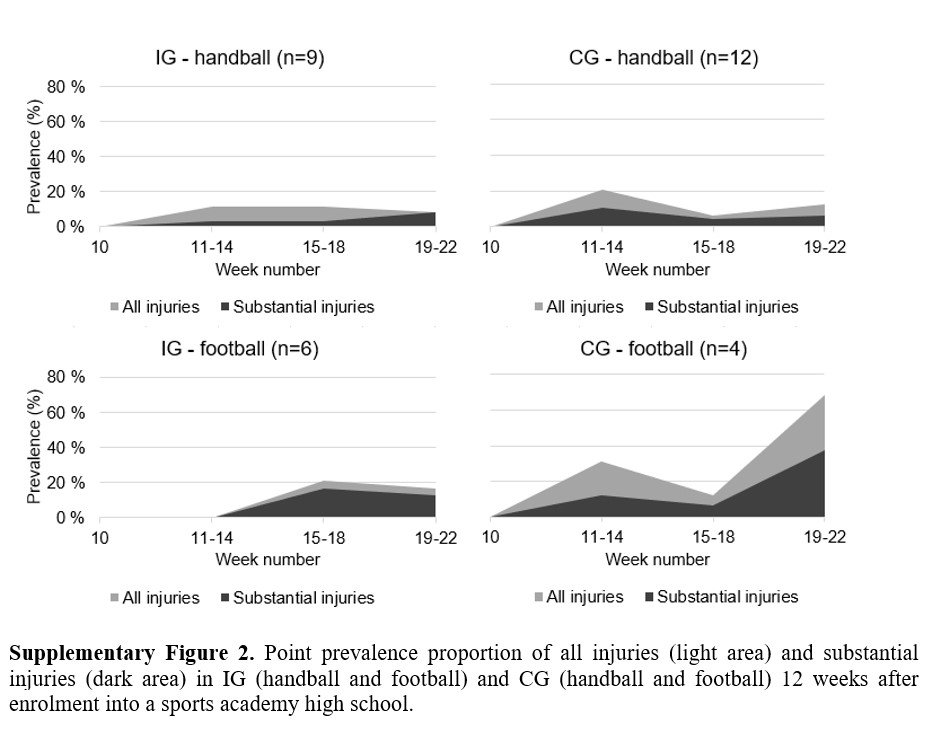

Supplement: Supplementary file 2 [file Image2.tiff]
